# Drug-Resistant Tuberculosis in Pet Ring-Tailed Lemur, Madagascar

**DOI:** 10.3201/eid2703.202924

**Published:** 2021-03

**Authors:** Marni LaFleur, Kim E. Reuter, Michael B. Hall, Hoby H. Rasoanaivo, Stuart McKernan, Paulo Ranaivomanana, Anita Michel, Marie Sylvianne Rabodoarivelo, Zamin Iqbal, Niaina Rakotosamimanana, Simon Grandjean Lapierre

**Affiliations:** Lemur Love Inc., San Diego, California, USA (M. LaFleur);; University of San Diego, San Diego (M. LaFleur, K.E. Reuter);; University of Utah, Salt Lake City, Utah, USA (K.E. Reuter);; European Bioinformatics Institute, Cambridge, UK (M.B. Hall, Z. Iqbal);; University of Antananarivo, Antananarivo, Madagascar (H.H. Rasoanaivo);; Wildlife One Health, Mtunzini, South Africa (S. McKernan);; Institut Pasteur de Madagascar, Antananarivo (P. Ranaivomanana, M.S. Rabodoarivelo, N. Rakotosamimanana, S. Grandjean Lapierre);; University of Pretoria, Pretoria, South Africa (A. Michel);; Université de Montréal, Montréal (S. Grandjean Lapierre)

**Keywords:** tuberculosis and other mycobacteria, lemur, Madagascar, illegal, pet, wildlife, lineage 3, lymph, antimicrobial resistance, anthropozoonotic, bacteria, TB, zoonoses

## Abstract

We diagnosed tuberculosis in an illegally wild-captured pet ring-tailed lemur manifesting lethargy, anorexia, and cervical lymphadenopathy. Whole-genome sequencing confirmed the *Mycobacterium tuberculosis* isolate belonged to lineage 3 and harbored streptomycin resistance. We recommend reverse zoonosis prevention and determination of whether lemurs are able to maintain *M. tuberculosis* infection.

Tuberculosis (TB) is an ancient disease affecting a plethora of domestic and wild animals, including humans. In primates, TB can cause severe multisystemic disease. The prevalence of TB in lemurs within Madagascar is unknown; the most recent documented case occurred in 1973 (*1*). Reverse zoonotic transmission of TB can occur when nonhuman primates are in close contact with humans (*1*). We report a clinical case and genomic analysis of TB infection in a female subadult ring-tailed lemur (*Lemur catta*) held at a nongovernmental organization facility in Southwestern Madagascar. The University of San Diego (San Diego, CA, USA) provided ethics authorization (no. IACUC 0619-01).

The lemur was born in the wild in September or October 2018 and was surrendered to the facility in April 2019. On July 12, the animal was emaciated, anorexic, and lethargic; it had a large fistulated mass on the left cervical region. The mass was surgically removed and found to be caseous and necrotic (Figure). Despite rehydration and systemic antimicrobial therapy, the lemur died on July 16.

**Figure:**
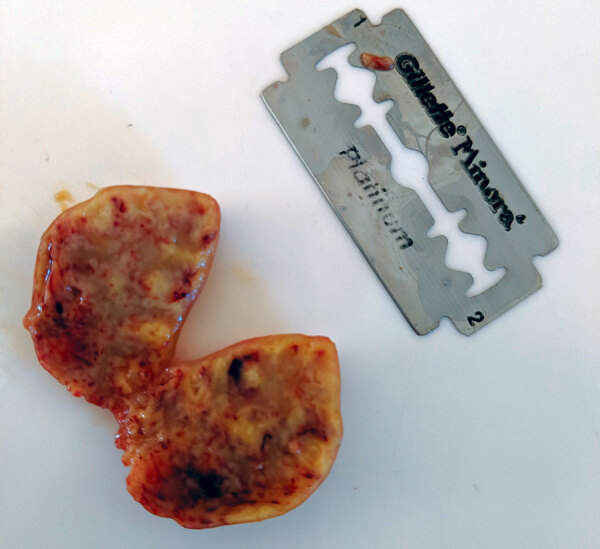
Lymph node removed from ring-tailed lemur in Madagascar that exhibited advanced clinical symptoms consistent with tuberculosis. Blade is 44 mm by 22 mm.

We confirmed TB infection by PCR on the lymph node sample using GeneXpert MTB/RIF assay (Cepheid, https://www.cepheid.com) (*2*). We cultured on Löwenstein-Jensen solid medium to confirm streptomycin resistance using the proportions method, enabling phenol chloroform DNA extraction and genomic DNA sequencing using Oxford Nanopore Technologies (ONT) (https://www.nanoporetech.com) long-read sequencing. We basecalled raw data using ONT Guppy software version 3.4.5. We performed read mapping using minimap2 version 2.17. For decontamination, we used a manually curated database including viral nontuberculosis mycobacteria and human sequences, augmented with *L. catta* genome (GenBank accession no. PVHV00000000) to improve host DNA filtering. Decontaminated reads were mapped to the *M. tuberculosis* H37Rv reference genome (accession no. NC_000962.3); we called single-nucleotide polymorphisms (SNPs) using bcftools version 1.10 (http://samtools.github.io/bcftools/bcftools.html) and masked repetitive regions (*3*). We performed genotypic resistance testing using Mykrobe Predict version 0.8.2 (https://www.mykrobe.com) and confirmed streptomycin resistance (causative variant R83P/CCG4407954CGG) (*4*). For lineage identification, we used both SNP-based method, which uses known lineage-defining SNPs, and k-mer-based methods, which rely on an in-silico equivalent of PCR probes analyzing each SNP alleles’ 20bp flanking regions (*5*–*7*). Both methods confirmed the isolate as lineage 3.1.1 (Central Asian sublineage Kilimanjaro, CASI-KILI). We ruled out laboratory cross-contamination and assessed relative genomic distance of this isolate compared with other human TB isolates from Madagascar by reanalyzing all lineage 3 TB isolates cultured in the laboratory during March 2017–June 2019 for which lineage typing and genomic sequencing data were available, and created a SNP distance matrix and phylogenetic tree (Appendix Figures 1, 2). The lemur’s isolate was substantially distant from other isolates by a closest SNP distance of 63 SNPs (mada_116), ruling out laboratory contamination or transspecies transmission within the samples processed on site (*3*). We submitted *M. tuberculosis* lineage 3 consensus sequence to GenBank (accession no. PRJNA659624).

Human TB isolates in the region of Toliara most frequently belong to lineage 1 (Institut Pasteur de Madagascar, unpub. data). However, lineage 3 isolates were previously isolated in humans from other regions of Madagascar (*7*). Pet lemurs are transported over vast distances (*8*,*9*); this lemur may have originated or been transferred from another region of Madagascar. The Malagasy lineage 3 profile shares similarities with strains found in Tanzania and the Indo-Pakistanese subcontinent (*7*).

Human activities, including trade and translocation of wild animals and keeping of wildlife as pets, have resulted in reverse zoonotic TB and spillover into wild populations in, among others, meerkats (*Suricata suricatta*), banded mongoose (*Mungos mungo*), and Asian elephants (*Elephas maximus*) (*10*). Although the risks of transmitting emerging diseases from wildlife to humans have received much attention, the risks that human diseases present to wildlife are not well described. In addition to other anthropogenic activities that imperil wildlife (e.g., deforestation, bushmeat consumption, animal trafficking), the effects of human disease reservoirs may become increasingly detrimental (*10*).

Illegal trade of wild-captured lemurs is rampant in Madagascar (*8*,*9*). Moreover, humans are frequently in close contact with pet or tourist facility–based lemurs. Some resorts encourage tourists to feed lemurs from their mouths, whereby pathogens could be transferred. Because lemurs make poor pets and often become aggressive, many are discarded as adults, some by release into forests with wild conspecific populations (*8*,*9*). 

We present anatomopathologic and molecular diagnostics evidence that wild-born lemurs can become infected with and die from complications of TB. To minimize risk for transmission of TB between humans and lemurs, we recommend enforced prohibition of keeping wild-captured lemurs as pets, systematic clinical screening and microbiological testing of facility-based animals and staff who become ill, and necropsy of deceased lemurs. Because we do not know if lemurs are able to maintain *M. tuberculosis* infection, we recommend quarantine and testing at lemur facilities and caution against release of captive lemurs into the wild. We also warn against close proximity or contact between humans and lemurs (captive or wild) in Madagascar, given the potential for reverse zoonotic and zoonotic transmission of TB and other infectious diseases.

Appendix Additional information about *M. tuberculosis* infection in a ring-tailed lemur, Madagascar.

## References

[1] Blancou J, Rakotoniaina P, Cheneau Y. Types of TB bacteria in humans and animals in Madagascar [in French]. Arch Inst Pasteur Madagascar. 1974;43:31–8.

[2] Ligthelm LJ, Nicol MP, Hoek KGP, Jacobson R, van Helden PD, Marais BJ, et al. Xpert MTB/RIF for rapid diagnosis of tuberculous lymphadenitis from fine-needle-aspiration biopsy specimens. J Clin Microbiol. 2011;49:3967–70. 10.1128/JCM.01310-11 21880965 PMC3209093

[3] Hunt M, Bradley P, Lapierre SG, Heys S, Thomsit M, Hall MB, et al. Antibiotic resistance prediction for *Mycobacterium tuberculosis* from genome sequence data with Mykrobe. Wellcome Open Res. 2019;4:191. 10.12688/wellcomeopenres.15603.1 32055708 PMC7004237

[4] Shitikov E, Kolchenko S, Mokrousov I, Bespyatykh J, Ischenko D, Ilina E, et al. Evolutionary pathway analysis and unified classification of East Asian lineage of *Mycobacterium tuberculosis.* Sci Rep. 2017;7:9227. 10.1038/s41598-017-10018-5 28835627 PMC5569047

[5] Rutaihwa LK, Menardo F, Stucki D, Gygli SM, Ley SD, Malla B, et al. Multiple introductions of *Mycobacterium tuberculosis* lineage 2–Beijing into Africa over centuries. Front Ecol Evol. 2019;7:112. 10.3389/fevo.2019.00112

[6] Stucki D, Brites D, Jeljeli L, Coscolla M, Liu Q, Trauner A, et al. *Mycobacterium tuberculosis* lineage 4 comprises globally distributed and geographically restricted sublineages. Nat Genet. 2016;48:1535–43. 10.1038/ng.3704 27798628 PMC5238942

[7] Ferdinand S, Sola C, Chanteau S, Ramarokoto H, Rasolonavalona T, Rasolofo-Razanamparany V, et al. A study of spoligotyping-defined *Mycobacterium tuberculosis* clades in relation to the origin of peopling and the demographic history in Madagascar. Infect Genet Evol. 2005;5:340–8. 10.1016/j.meegid.2004.10.002 16168940

[8] LaFleur M, Clarke TA, Reuter KE, Schaefer MS, terHorst C. Illegal trade of wild-captured *Lemur catta* within Madagascar. Folia Primatol (Basel). 2019;90:199–214. 10.1159/000496970 31067551

[9] Reuter KE, LaFleur M, Clarke TA, Holiniaina Kjeldgaard F, Ramanantenasoa I, Ratolojanahary T, et al. A national survey of household pet lemur ownership in Madagascar. PLoS One. 2019;14:e0216593. 10.1371/journal.pone.0216593 31067269 PMC6506143

[10] Chomel BB, Belotto A, Meslin FX. Wildlife, exotic pets, and emerging zoonoses. Emerg Infect Dis. 2007;13:6–11. 10.3201/eid1301.060480 17370509 PMC2725831

