# Correction: Vol. 27, No. 3

**DOI:** 10.3201/eid2705.C12705

**Published:** 2021-05

**Keywords:** errata, erratum, correction

The order of the authors was incorrect for Drug-Resistant Tuberculosis in Pet Ring-Tailed Lemur, Madagascar (M. LaFleur et al.). The article has been corrected online (https://wwwnc.cdc.gov/eid/article/27/3/20-2924_article).

