# Supplementary material for: Drug-Resistant Tuberculosis in Pet Ring-Tailed Lemur, Madagascar
Source: Emerg Infect Dis. 2021 Mar;27(3):977–9. doi: 10.3201/eid2703.202924 (PMC7920673; doi:10.3201/eid2703.202924)
Supplement: Appendix — Additional information about M. tuberculosis infection in a ring-tailed lemur, Madagascar. [file 20-2924-Techapp-s1.pdf]

Article DOI: https://doi.org/10.3201/eid2703.202924

Drug-Resistant Tuberculosis in Pet Ring-Tailed Lemur, Madagascar

Appendix

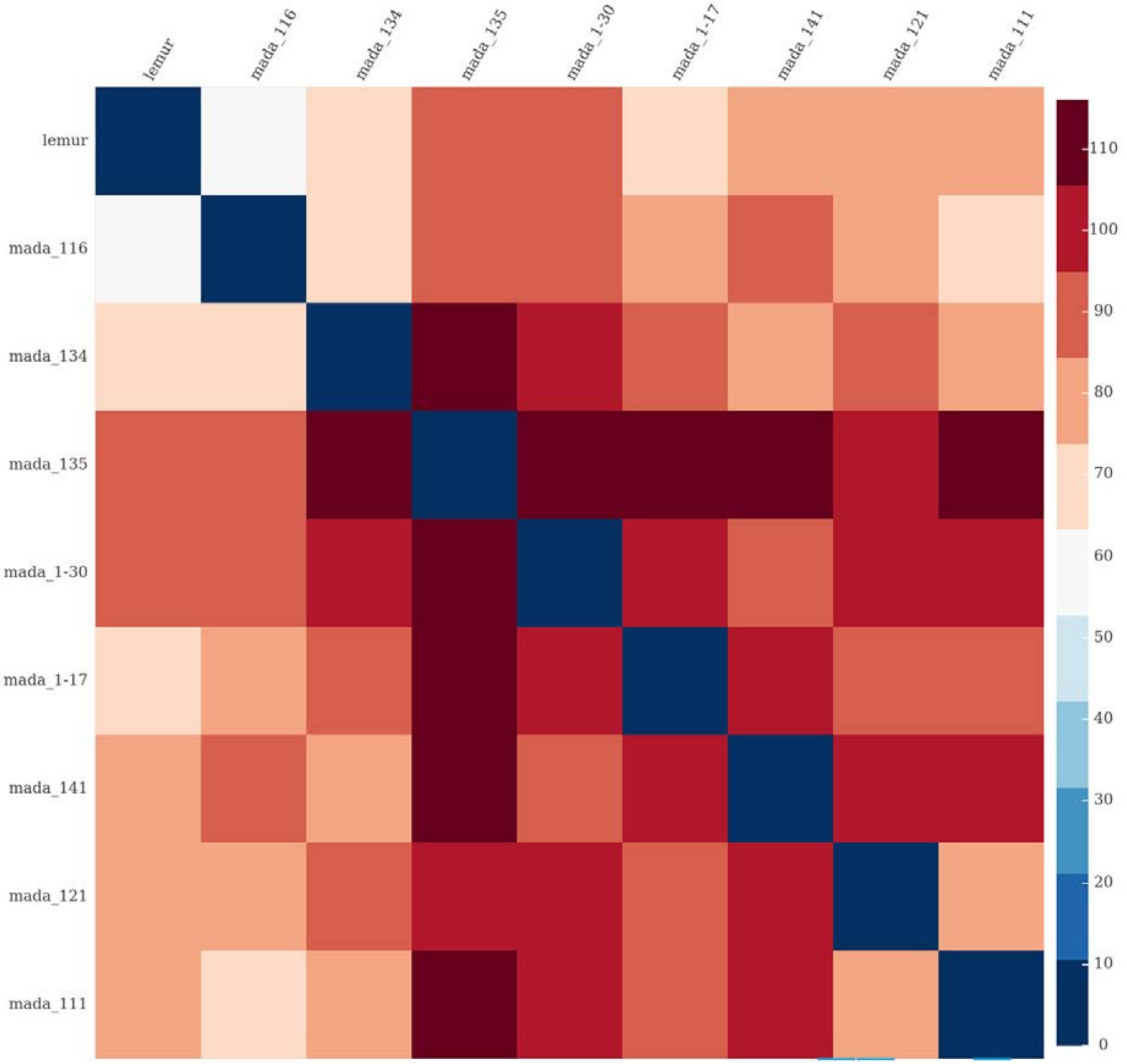

Appendix Figure 1. Heatmap showing pairwise SNP distance between a lemur *Mycobacterium tuberculosis* isolate and other human lineage 3 samples based on genomic DNA sequencing and SNP calling (Oxford Nanopore Technologies; https://www.nanoporetech.com). Colors indicate distance; blue is closer and red is more distant.

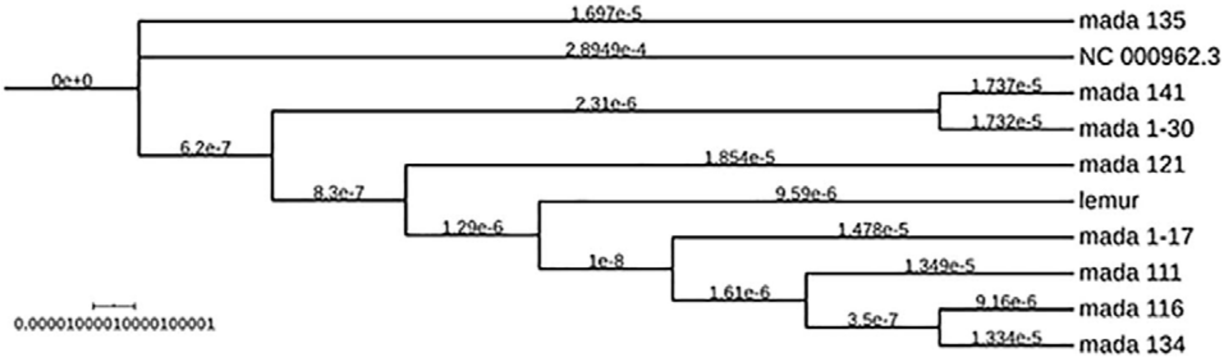

Appendix Figure 2. Phylogenetic tree of lemur *Mycobacterium tuberculosis* isolate, other human lineage 3 samples, and *Mycobacterium tuberculosis* H37Rv lineage 4 reference sequence (GenBank accession no. NC_000962.3) PhyML was used to construct the tree. Numbers on branches indicate the length of the branch; scale bar indicates average number of nucleotide substitutions per site.
